# PU.1 regulates Alzheimer’s disease-associated genes in primary human microglia

**DOI:** 10.1186/s13024-018-0277-1

**Published:** 2018-08-20

**Authors:** Justin Rustenhoven, Amy M. Smith, Leon C. Smyth, Deidre Jansson, Emma L. Scotter, Molly E. V. Swanson, Miranda Aalderink, Natacha Coppieters, Pritika Narayan, Renee Handley, Chris Overall, Thomas I. H. Park, Patrick Schweder, Peter Heppner, Maurice A. Curtis, Richard L. M. Faull, Mike Dragunow

**Affiliations:** 1 Department of Pharmacology and Clinical Pharmacology, The University of Auckland, Private Bag 92019, Auckland, 1142 New Zealand; 2 Centre for Brain Research, The University of Auckland, Auckland, New Zealand; 3 Division of Brain Sciences, Department of Medicine, Imperial College London, London, UK; 4 Department of Anatomy and Medical Imaging, The University of Auckland, Auckland, New Zealand; 5 School of Biological Sciences, The University of Auckland, Auckland, New Zealand; 6 Center for Brain Immunology and Glia, University of Virginia, Charlottesville, Virginia USA; 7 Departmemt of Neuroscience, University of Virginia, Charlottesville, Virginia USA; 8 Auckland City Hospital, Auckland, New Zealand

**Keywords:** Alzheimer’s disease, Vorinostat, Phagocytosis, Antigen presentation, Drug screening, Neuroinflammation

## Abstract

**Background:**

Microglia play critical roles in the brain during homeostasis and pathological conditions. Understanding the molecular events underpinning microglial functions and activation states will further enable us to target these cells for the treatment of neurological disorders. The transcription factor PU.1 is critical in the development of myeloid cells and a major regulator of microglial gene expression. In the brain, PU.1 is specifically expressed in microglia and recent evidence from genome-wide association studies suggests that reductions in PU.1 contribute to a delayed onset of Alzheimer’s disease (AD), possibly through limiting neuroinflammatory responses.

**Methods:**

To investigate how PU.1 contributes to immune activation in human microglia, microarray analysis was performed on primary human mixed glial cultures subjected to siRNA-mediated knockdown of PU.1. Microarray hits were confirmed by qRT-PCR and immunocytochemistry in both mixed glial cultures and isolated microglia following PU.1 knockdown. To identify attenuators of PU.1 expression in microglia, high throughput drug screening was undertaken using a compound library containing FDA-approved drugs. NanoString and immunohistochemistry was utilised to investigate the expression of PU.1 itself and PU.1-regulated mediators in primary human brain tissue derived from neurologically normal and clinically and pathologically confirmed cases of AD.

**Results:**

Bioinformatic analysis of gene expression upon PU.1 silencing in mixed glial cultures revealed a network of modified AD-associated microglial genes involved in the innate and adaptive immune systems, particularly those involved in antigen presentation and phagocytosis. These gene changes were confirmed using isolated microglial cultures. Utilising high throughput screening of FDA-approved compounds in mixed glial cultures we identified the histone deacetylase inhibitor vorinostat as an effective attenuator of PU.1 expression in human microglia. Further characterisation of vorinostat in isolated microglial cultures revealed gene and protein changes partially recapitulating those seen following siRNA-mediated PU.1 knockdown. Lastly, we demonstrate that several of these PU.1-regulated genes are expressed by microglia in the human AD brain in situ.

**Conclusions:**

Collectively, these results suggest that attenuating PU.1 may be a valid therapeutic approach to limit microglial-mediated inflammatory responses in AD and demonstrate utility of vorinostat for this purpose.

**Electronic supplementary material:**

The online version of this article (10.1186/s13024-018-0277-1) contains supplementary material, which is available to authorized users.

## Background

Microglia are the resident macrophages in the central nervous system (CNS) and primary mediators of neuroinflammation. Whilst they are beneficial in pruning unnecessary synapses during development [1] and removing pathogens and cellular debris [2], microglia can also contribute to neurological dysfunction [3]. Elevated microglial-mediated neuroinflammatory responses can perturb neuronal functioning or induce neuronal death [4], and unrestricted phagocytosis of stressed-but-viable cells can lead to inappropriate removal of neurons via phagoptosis [5]. Numerous stimuli can promote microglial inflammatory responses, including microorganism recognition, peripheral inflammation, CNS damage, as well as recognition of misfolded proteins, including amyloid-beta (Aβ) plaques present in Alzheimer’s disease (AD) [3, 6].

AD is a progressive neurodegenerative disorder characterized symptomatically by gradual memory impairment and other cognitive deficits [7]. Pathologically, the AD brain displays extensive extracellular deposition of parenchymal Aβ plaques and intracellular neurofibrillary tangles composed of hyperphosphorylated tau [8]. It is well recognized that elevated microglia inflammatory responses precipitated by these misfolded proteins contribute to disease progression [9]. Importantly, microglial inflammatory responses are not unique to AD but are present, and indeed detrimental, in other neurodegenerative disorders (Parkinson’s disease [10], Huntington’s disease [11], amyotrophic lateral sclerosis [12], and multiple sclerosis [13]), epilepsy, neuropsychiatric disorders (depression [14] and autism [15]), and acute brain injuries (stroke [16] and traumatic brain injuries [17]), making microglia an attractive therapeutic target.

Whilst the exact cause of AD remains elusive, there is a significant genetic component. Approximately 1–6% of all cases are classed as early-onset AD, typically with mutations in amyloid-processing genes (*APP*, *PSEN1* and *PSEN2*) [18], whilst genome wide-association studies (GWAS) have identified numerous risk variant genes associated with late onset Alzheimer’s disease (AD) [19–28]. Interestingly, several of these genes, including *TREM2*, *CD33*, *ABCA7*, *HLA-DRB5*, and *MS4A4,* are highly expressed by myeloid cells and are involved in innate and adaptive immune mechanisms [29–31]. These findings have helped shape our understanding of the neuroinflammatory component of this disease. Recently, a genome-wide survival analysis identified a common haplotype, rs1057233^g^, in the *CELF1* AD risk locus which displayed reduced expression of PU.1 in monocytes and macrophages and delayed age of onset of AD [32]. The transcription factor PU.1 (*SPI1*) is a master regulator of myeloid cells and controls microglial development and function [33, 34]. In the CNS its expression is limited to microglia and PU.1 knockdown or overexpression in the BV2 rodent microglia cell line identified a hub of differentially expressed genes relating to neuroimmune responses [32]. Additionally, mutant Huntingtin aggregates present in Huntington’s disease enhance microglial activation through PU.1 [35], as do hypoxic-ischaemic insults [36], suggesting that PU.1 modulation may be a common feature underlying distinct neurological disorders. Further, overexpression of the CNS-enriched miRNA124 [37] attenuates macrophage inflammatory responses through reduced C/EBPα and PU.1 signalling [38], as well as preventing CNS inflammation and associated epileptogenesis [39]. As such, attenuation of PU.1 may be a valid therapeutic strategy to limit microglia-mediated neuroinflammation in various neurological disorders.

Whilst there is overwhelming evidence implicating microglial gene variants in modifying AD risk [31], exactly how these variants contribute to human disease is uncertain. Current studies concerning PU.1 have been performed solely using in vitro/in vivo rodent models or in vitro human macrophages and whether these findings translate to primary human microglia remains unclear. Human microglia display significant species differences from their more frequently used rodent counterparts [40, 41] and several of the highly differentially expressed genes are risk factors for neurodegenerative diseases, particularly AD [30]. Additionally, RNAseq analysis of isolated microglia and macrophages in mice revealed a distinct gene signature in microglia with differential expression of innate immune system genes [42], whilst macrophages and microglia also differ in their inflammatory profile in acute ischaemia [43]. Additionally, treatment with the histone deacetylase (HDAC) inhibitor valproic acid (VPA) prevented phagocytosis of Aβ_1–42_ by human microglia [66], whilst the reverse was true for the rodent microglial BV2 cell line [44], further necessitating caution when identifying pharmacological agents to modify microglial functions in human disease [40, 45]. Together, such findings suggest caution when extrapolating information obtained from non-human microglia.

Previously, we have demonstrated human microglial expression of PU.1, both in vitro and in situ, and its attenuation was found to prevent microglial phagocytosis of Aβ_1–42_ [33]. Here we sought mechanistic insights into the role of PU.1 in regulating immune functions in primary human microglia. We identified changes in innate and adaptive immune pathways, particularly genes involved in antigen presentation and phagocytosis, and confirmed these changes in isolated microglial cultures. Utilising high throughput screening of 1280 FDA approved compounds in primary human brain mixed glial cultures, we identified the HDAC inhibitor vorinostat as a candidate drug for attenuating PU.1 expression in human microglia and mimicking several of the effects of PU.1 silencing. Finally, we demonstrate that several of these PU.1-regulated genes are expressed and upregulated by microglia in the human AD brain and suggest that modulating PU.1 expression may be a valid therapeutic target to prevent microglial-mediated neurodegeneration.

## Methods

### Tissue source

For primary human cell culture and subsequent in vitro studies, human brain tissue was obtained, with informed written patient consent, from various sources of neurosurgical tissue (Additional file 1: Table S1). For imummunohistochemical studies, middle temporal gyrus (MTG) from post mortem adult human brain tissue of neurologically normal or clinically and pathologically confirmed cases of AD (Additional file 1: Table S1) was obtained from the Neurological Foundation Douglas Human Brain Bank, processed as described previously [46]. All protocols used in this study were approved by the Northern Regional Ethics Committee (New Zealand) for biopsy tissue and the University of Auckland Human Participants Ethics Committee (New Zealand) for post mortem tissue. All donors underwent a full consent process. All methods were carried out in accordance with the approved guidelines.

### Cell isolation and culture

Mixed glial cultures containing astrocytes, pericytes, endothelial cells, and microglia were isolated from human brain tissue as described previously [47] and used at passage two. Isolated pericyte cultures were generated from these initial mixed glial cultures by subsequent passaging in order to dilute out non-proliferating microglia, astrocytes, and endothelial cells as described previously [48]. Isolated microglial cultures were generated as described previously [49]. Cells were harvested using 0.25% trypsin- 1 mM EDTA (Gibco, CA, USA) with mixed glial cultures and microglia cultures also utilising gentle detachment with a cell scraper (Falcon, MA, USA) due to strong microglial attachment. Viability was determined by trypan blue exclusion (Gibco). Mixed glial and pericyte cultures were plated at 15,000 cells/cm^2^ and isolated microglia were plated at 30,000 cells/cm^2^ in Nunc™ microwell plates with Nunclon™ Delta surface (Nunc, Denmark). All cultures were maintained in DMEM/F12 (Gibco), 10% fetal bovine serum (FBS; Moregate, Australia) and 1% penicillin streptomycin glutamine (PSG; Gibco).

### siRNA transfection

Cells were transfected with 50 nM PU.1 (*SPI1*) specific siRNA (SASI_Hs02_00315880; Sigma Aldrich, MO, USA) or a non-targeting siRNA sequence (Universal Negative Control #1) using Lipofectamine™ RNAiMAX (Life Technologies, CA, USA). Cells were cultured for a further 7 days to allow for PU.1 knockdown with a full media change performed 48 h post-transfection. This procedure has previously been shown to generate efficient reduction of PU.1 expression in human brain microglia [33].

### Immunocytochemistry and fluorescent microscopy

Cells were fixed for 15 min using 4% paraformaldehyde (Scharlau, Spain) and washed three times in phosphate buffered saline (PBS) with 0.1% Triton™ X-100 (PBS-T; Sigma Aldrich). Cells were incubated with primary antibodies (Additional file 2: Table S2) diluted in goat immunobuffer (1% goat serum (Gibco), 0.2% triton X-100 and 0.04% thiomersal (Sigma Aldrich) in PBS) at 4 °C overnight, washed three times in PBS-T and incubated with appropriate anti-species fluorescently conjugated secondary antibodies diluted in goat immunobuffer at 4 °C overnight. Cells were washed again in PBS-T and nuclei were counterstained with 20 nM Hoechst 33258 (Sigma Aldrich) for 20 min at room temperature. Images were acquired at 20 x magnification using the ImageXpress® Micro XLS automated fluorescent microscope (Version 5.3.0.1, Molecular Devices, CA, USA). Quantitative analysis of intensity measures and scoring of positively stained cells was performed using the Cell Scoring and Show Region Statistics analysis modules within MetaXpress® software (Molecular Devices). For analysis of microglial morphology by immunocytochemistry (ICC), CD45 staining was thresholded and the Integrated Morphometry Analysis tools Elliptical Form Factor (elongation factor; length/breadth) and Shape Factor (roundness factor; 4πA/P2, *P* = cell perimeter, A = cell area) were used to determine cell shape.

### Cytometric bead array

Conditioned media was collected from cells in 96-well plates, centrifuged for 5 min at 300 x *g* and the clarified supernatant was stored at − 20 °C. Cytokine concentrations were determined using a multiplexed cytometric bead array (CBA; BD Biosciences, CA, USA) as per the manufacturer’s instructions. Data was analysed using FCAP array™ software (version 3.1; BD Biosciences) to convert raw fluorescent values into concentrations using an 11-point standard curve (0–10,000 pg/mL).

### High throughput drug screening

High throughput screening to identify compounds which modify PU.1 expression was performed using a Chemical Library (Prestwick Chemical, France) containing 1280 small molecules consisting of mostly FDA approved drugs. Compounds were screened at 10 μM for 48 h in mixed glial cultures before fixing and immunostaining for PU.1 and counterstaining with Hoechst 33258. Cells were imaged using the ImageXpress® Micro XLS automated fluorescent microscope and the percentage of PU.1-positive cells and total Hoechst-positive cells was quantified, allowing alterations in PU.1 expression as well as total cell viability to be assessed. The histone deacetylase (HDAC) inhibitor vorinostat (also known as SAHA) was identified by this screen as an attenuator of PU.1 expression. Validation in two further mixed glial cultures confirmed this effect.

### Vorinostat treatment

For subsequent experiments cells were treated with 10 μM vorinostat (Sigma Aldrich) or a vehicle control (0.1% DMSO; Sigma Aldrich) for 24 h.

### RNA extraction and cDNA synthesis

For mixed glial and pericyte cultures, RNA was extracted using a TRIzol™(Invitrogen)/chloroform procedure followed by isolation using the RNeasy Mini Kit (Qiagen, Netherlands) as described previously [50]. For isolated microglia samples the RNAqueous™ Micro Total RNA Isolation kit (Ambion, CA, USA) was used to allow for efficient extraction from a small number of cells. For microarray samples, RNA quality was assessed using an Agilent 2100 bioanalyzer (Agilent Technologies, CA, USA) and all samples had RIN values of 10. For all samples, RNA concentration was determined using a Nanodrop (Thermo Fisher). All samples were treated with DNase I (1 μg DNase/1 μg RNA) using the RQ1 RNase-free DNase kit (Promega, WI, USA) and cDNA was prepared using the Superscript® III First-Strand Synthesis kit (Life Technologies).

### qRT-PCR

Quantitative real-time PCR (qRT-PCR) was performed using Platinum® SYBR® Green qRT-PCR SuperMix-UDG with Rox (Life Technologies, CA, USA) on a 7900HT Fast Real-Time PCR system (Applied Biosystems, CA, USA). Standard curves were run for all primers and efficiencies were all 100 ± 10% (Additional file 3: Table S3). Relative gene expression analysis was performed using the 2^-ΔΔCt^ method with the housekeeping gene *GAPDH* as described previously [51].

### Microarray and bioinformatics analysis

RNA was labelled and hybridised to Affymetrix Genechip® Primeview™ Human Gene Expression Arrays (Santa Clara, CA, USA) according to manufacturer’s instructions. Microarray was performed and analysed by New Zealand Genomics Limited (NZGL). Bioinformatics analysis was carried out in the ‘R’ statistical environment as described previously [50]. Briefly, the “.cel” files from each genechip were quality assessed using the ‘AffyQCReport’ package and were normalised using the RMA algorithm with background correction. To generate a list of differentially expressed genes, statistical analysis of gene abundance between samples was performed on log_2_ transformed data using the LIMMA method. The main queries were identifying differentially expressed genes in PU.1 siRNA samples relative to scrambled siRNA samples in mixed glial cultures and pericyte-only cultures. A list of 180 differentially expressed genes in mixed glial cultures with PU.1 siRNA compared to control siRNA was generated with fold changes > 1.5 and adjusted *p*-values < 0.001 (Additional file 4: Table S4). Of these 180 genes, 32 were excluded as they also showed fold changes of > 1.5 in pericyte only cultures with PU.1 siRNA relative to scrambled siRNA and were deemed off-target effects. A further 46 genes had multiple genes yielding 102 unique genes recognized using the Database for Annotation, Visualisation and Integrated Discovery (DAVID) gene list conversion. Relationships between the remaining 102 differentially expressed genes were further explored using DAVID (https://david.ncifcrf.gov/) gene ontology tools, including Biological Processes, Cellular components, and Molecular Functions. KEGG pathway analysis was performed in DAVID to determine biological pathway maps which were altered with PU.1 silencing yielding 16 pathways with *p*-values < 0.001. Further, STRING (https://string-db.org/) was utilised to investigate protein-protein interaction networks of PU.1 regulated genes. PU.1 (*SPI1*) was not in the list of 102 differentially regulated genes but was included in the STRING analysis for interaction purposes. For heatmap generation including unbiased hierarchical clustering the heatmap.2 function was utilised in ‘R’.

### NanoString

RNA was extracted from fresh frozen human brain tissue taken from the middle frontal gyrus of post-mortem control (*n* = 8) and AD (*n* = 8) cases (Additional file 1: Table S1). Care was taken to dissect only grey matter from the tissue samples (< 30 mg), which were then immediately homogenized in 1 mL TRIzol reagent with 2 mm stainless steel beads (Qiagen) using the Tissuelyser II tissue homogenizer (Qiagen) for 4 min at 25 Hz. Samples were centrifuged at 12,000 x *g* for 2 min at 4 °C. The supernatant was collected and 200 μL of chloroform added, shaken vigorously for 15 s and incubated for 2–3 min at RT and then centrifuged at 12,000 x g for 15 min at 4 °C. The upper aqueous phase was collected and mixed with an equal volume of 70% ethanol. Subsequent steps were performed using the RNeasy kit (Qiagen) following manufacturer’s instructions. DNase I treatment was performed using components from the RNAqueous-Micro kit (Ambion) following manufacturer’s instructions. RNA purity and concentrations were determined using Qubit and Bioanalyzer 2100 (Agilent Technologies). Only samples with RIN > 5 were used for Nanostring. Samples were shipped on dry ice to the Otago Genomics Facility (Otago, NZ) for further QC and processing on the Nanostring N-Counter using a custom CodeSet that included 5 reference housekeeping genes (*ACTB*, *PGK1*, *POL1B*, *RPLP0*, and *RPL30*) which were used for normalisation. All data passed QC, with no imaging, binding, positive control, or CodeSet content normalisation flags. Background-corrected counts (mean + 1SD) normalised to the geometric mean of both the positive controls (between lane hyb effects) and all nominated reference, housekeeping genes (RNA input effects) for all samples were used for graphs.

### Immunohistochemistry and fluorescent microscopy

Imummunohistochemistry (IHC) procedures were performed using free-floating, formalin fixed, 50 μm thick tissue sections, processed as described previously [46]. TSA™ SuperBoost™ kits (ThermoFisher) were used to amplify signal from TREM2 and DAP12 stains as per manufacturer’s instructions, while IBA1, HLA-DR,DP,DQ, and CD45 stains were performed using traditional primary-secondary labelling. Heat-induced epitope retrieval was performed in Tris-EDTA (ethylenediamine tetraacetic acid, 1 mM; Tris-HCl, 10 mM, pH 9). Endogenous peroxidase activity was then blocked (1% hydrogen peroxide, 50% methanol), before sections were incubated with primary antibody (72 h, 4 °C) diluted in 1% normal donkey serum (Gibco) in PBS-T. Sections were washed and incubated with fluorescent and biotinylated secondary antibodies raised in donkey (24 h, 4 °C) diluted in 1% normal donkey serum in PBS-T. The following day, sections were washed and incubated with extravidin peroxidase, diluted in 1% normal donkey serum in PBS-T, for 4 h at room temperature. Sections were washed, and tyramide reaction solution (tyramide-488, 1:500; hydrogen peroxide, 0.03% in reaction buffer) added for 15 min at room temperature. The reaction was quenched using stop solution, and Hoechst 33342 nuclear counterstain added (1:10,000, 5 min; Thermo Fisher). Tissue sections were imaged at 20 x magnification using a Nikon Eclipse Ni microscope (Japan). For each section, two 2.00 × 2.00 mm regions were imaged from grey and white matter across two distant sections were imaged per case (*n* = 5 control, *n* = 4 AD).

### Statistical analysis

All cell culture experiments were performed at least three independent times on tissue from three different donors. Statistical analysis was performed using an unpaired Students *t* test, or a two-way ANOVA with Tukey’s *post-hoc* multiple comparison test, as designated in figure legends (Graphpad Prism 7, CA, USA). Statistical analysis of microarray data was performed as previously described [50]. All data is displayed as mean+/− SEM.

## Results

### Characterisation of culture conditions for microarray analysis

**Fig. 1:**
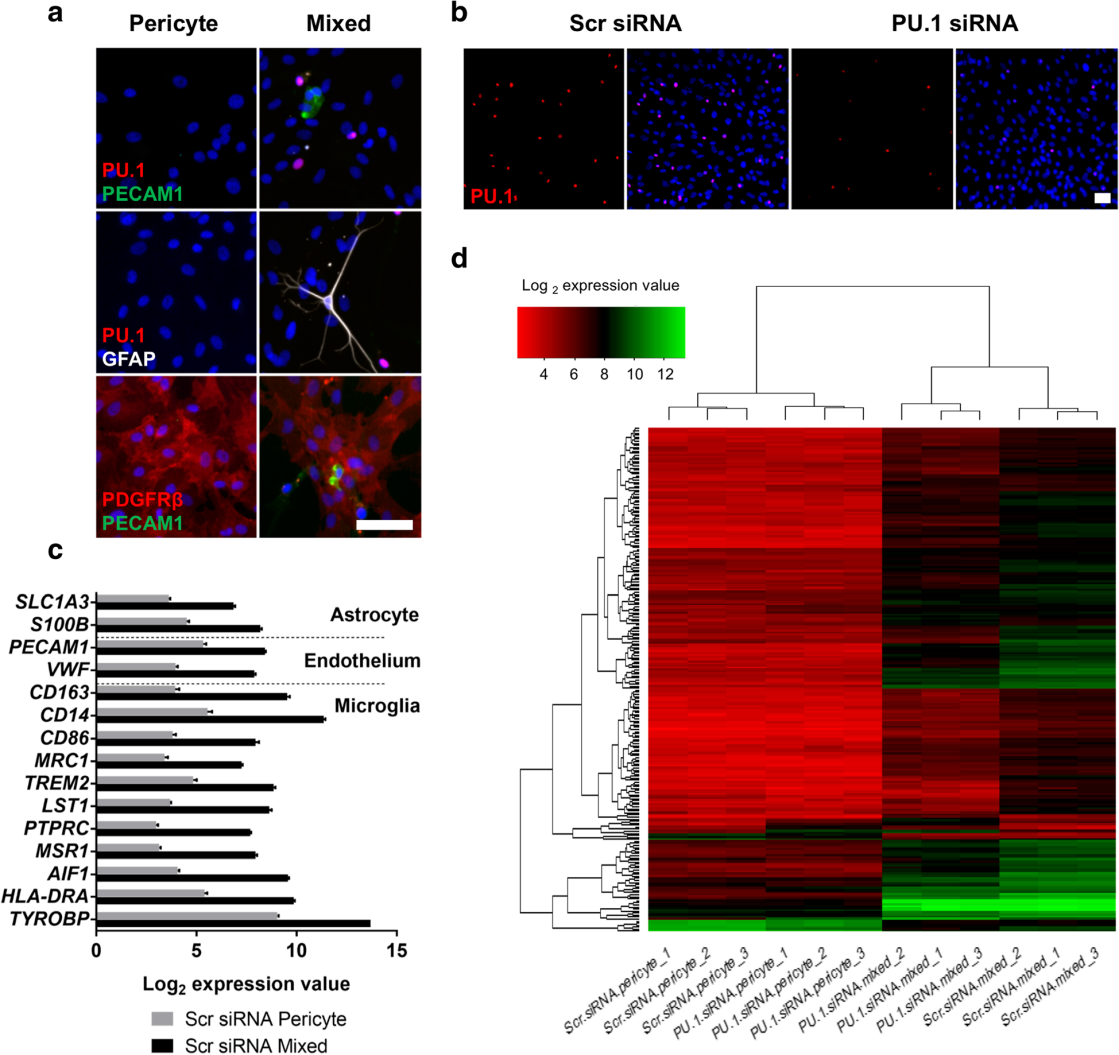
Characterisation of culture conditions for microarray analysis. **a** Characterisation of primary human mixed glial cultures comprised of pericytes (PDGFRβ), microglia (PU.1), astrocytes (GFAP), and endothelial cells (PECAM1), compared with pericyte-only cultures containing PDGFRβ only, scale bar = 100 μm. **b** PU.1 knockdown in mixed glial cultures transfected with 50 nM scrambled or PU.1 siRNA for 7 days, scale bar = 100 μm. **c** Microarray analysis of cell specific genes in scrambled siRNA transfected mixed glial cultures or pericyte-only cultures. **d** Unbiased hierarchical clustering of the top 250 differentially expressed genes from microarray analysis of mixed glial cultures or pericyte cultures transfected with 50 nM scrambled or PU.1 siRNA for 7 days, *n* = 3 independent microglial cultures

Characterisation of mixed glia isolated from human brain biopsy tissue revealed heterogeneous cultures containing microglia (PU.1), endothelia (PECAM1), astrocytes (GFAP), and pericytes (PDGFRβ; Fig. 1a). In contrast, pericyte cultures displayed PDGFRβ expression whilst lacking PU.1, GFAP, and PECAM1 (Fig. 1a). Transfection of mixed glial cultures with PU.1 siRNA was effective in attenuating microglial PU.1 expression compared to a scrambled siRNA (Fig. 1b), as described previously [33]. As expected, microarray analysis revealed a higher expression of astrocyte (*SLC1A3* and *S100B*), endothelial (*PECAM1* and *VWF*), and microglial (*CD163*, *CD14*, *CD86*, *MRC1*, *TREM2*, *LST1*, *PTPRC*, *MSR1*, *AIF1*, *HLADRA*, and *TYROBP*) genes in scrambled siRNA transfected mixed glial cultures compared to similarly treated pericyte-only cultures (Fig. 1c), reflecting the immunocytochemistry analysis (Fig. 1a). Unbiased hierarchical clustering of the top 250 differentially expressed genes across all samples revealed the similarity of biological replicates obtained from different tissue donors. Further, numerous changes were evident in PU.1 siRNA-transfected samples compared to scrambled siRNA in mixed glial cultures, containing PU.1^+^ microglia, whilst little change was observed in pericyte-only cultures (Fig. 1d).

### Microarray analysis of PU.1 silencing

**Fig. 2:**
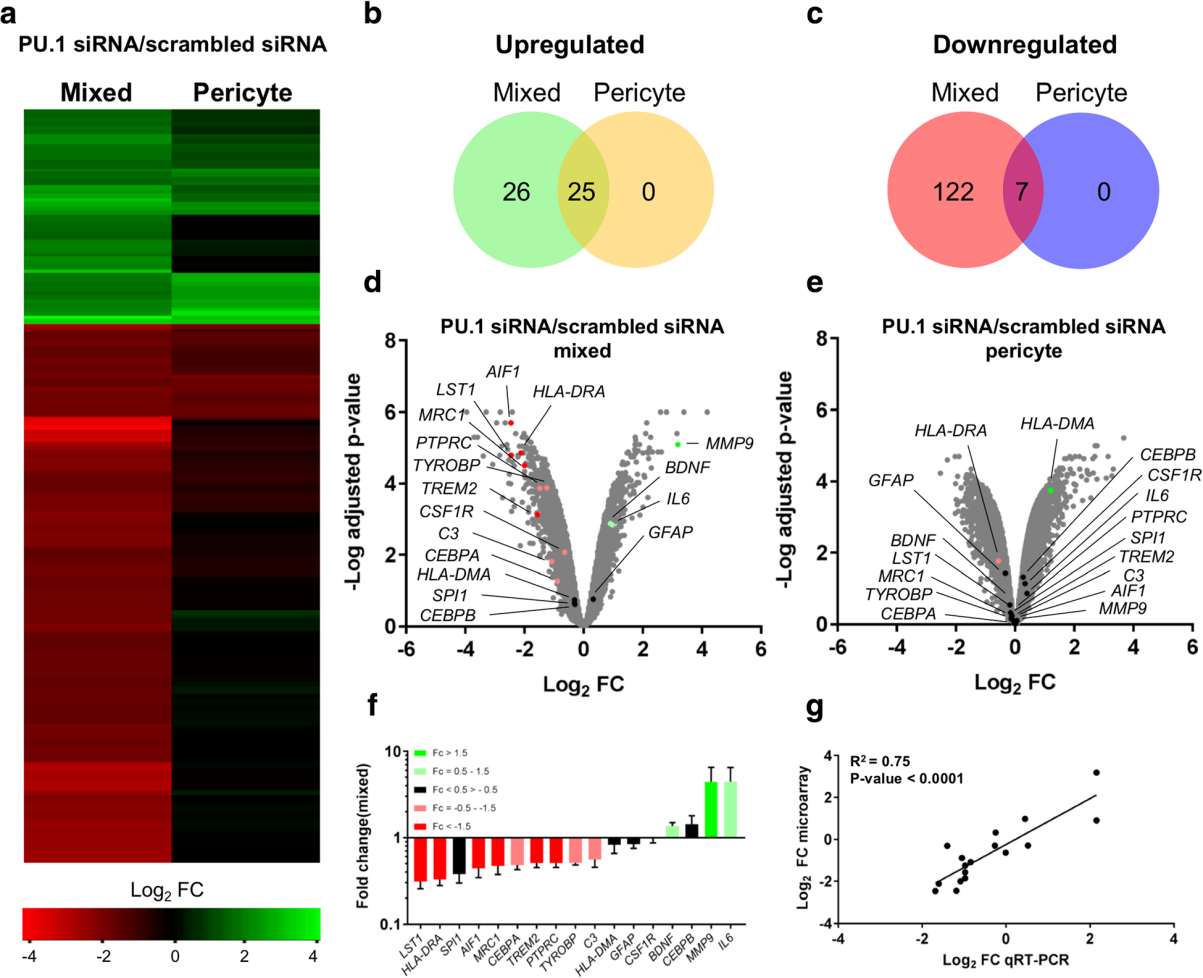
Microarray analysis of PU.1 silencing. **a** Heatmap generation of the top 180 differentially expressed genes (log_2_ fold change > 1.5, adjusted *p*-value < 0.001) with PU.1 siRNA versus control siRNA in mixed glial cultures. Corresponding changes in pericyte-only cultures are shown. **b**, **c** Venn diagrams revealing 51 upregulated genes by PU.1 silencing, 26 of which were specific to mixed glial cultures and 129 downregulated genes, 122 of which were specific to mixed glial cultures. **d**, **e** Volcano plots displaying log_2_ fold change versus –log adjusted p-value for all genes (grey) and genes of interest displaying significant reduction (dark red), mild reductions (light red), no change (black), mild induction (light green), or significant induction (dark green) in mixed glial cultures or pericyte only cultures. **f** Validation of selected genes by qRT-PCR (*n* = 3 independent microglial cultures) in mixed glial cultures transfected with 50 nM PU.1 siRNA versus control siRNA for 7 days, colour coded by their significance from microarray analysis. Data is displayed as fold change of mRNA genes in PU.1 siRNA treated cultures relative to control siRNA samples as determined by the 2^^-ΔΔCt^ method (**g**) Log_2_ fold changes between microarray and qRT-PCR analysis demonstrated good correlation (R^2^ = 0.75)

In order to investigate the effect of PU.1 knockdown in mixed glial cultures by microarray analysis, gene expression in PU.1 siRNA samples was normalised to scrambled siRNA samples in either mixed glial cultures or pericyte-only cultures. The top 180 differentially expressed genes displaying log_2_ fold changes > 1.5 and adjusted *p*-values < 0.001 were selected for further analysis (Additional file 4: Table S4), and the corresponding changes in pericyte-only cultures were determined to identify potential off-target effects of PU.1 siRNA (Fig. 2a). Of the 180 differentially expressed genes, 51 were found to be upregulated in both the pericyte and mixed glial cultures, with 26 uniquely upregulated genes in mixed glial cultures (Fig. 2b). Similarly, 129 downregulated genes were identified, 7 of which were present in both culture conditions revealing 122 uniquely downregulated genes (Fig. 2c). A panel of 17 mostly microglial-specific genes (*LST1*, *HLA*-*DRA*, *SPI1*, *AIF1*, *MRC1*, *CEBPA*, *TREM2*, *PTPRC*, *TYROBP*, *C3, HLA*-*DMA*, *GFAP*, *CSF1R*, *BDNF*, *CEBPB*, *MMP9*, and *IL6*) displaying large reductions (log_2_ FC > − 1.5), moderate reductions (log_2_ FC = − 0.5 - -1.5), no change (log_2_ FC = − 0.5 – 0.5), small inductions (log_2_ FC = 0.5–1.5), and large inductions (log_2_ FC > 1.5) in mixed glial cultures were selected (Fig. 2d,e) and validated by qRT-PCR (Fig. 2f) to confirm microarray findings, with gene expression showing good correlation (R^2^ = 0.75) between methods (Fig. 2g).

### Bioinformatic analysis reveals PU.1 as a highly connected hub protein involved in innate and adaptive immunity

**Fig. 3:**
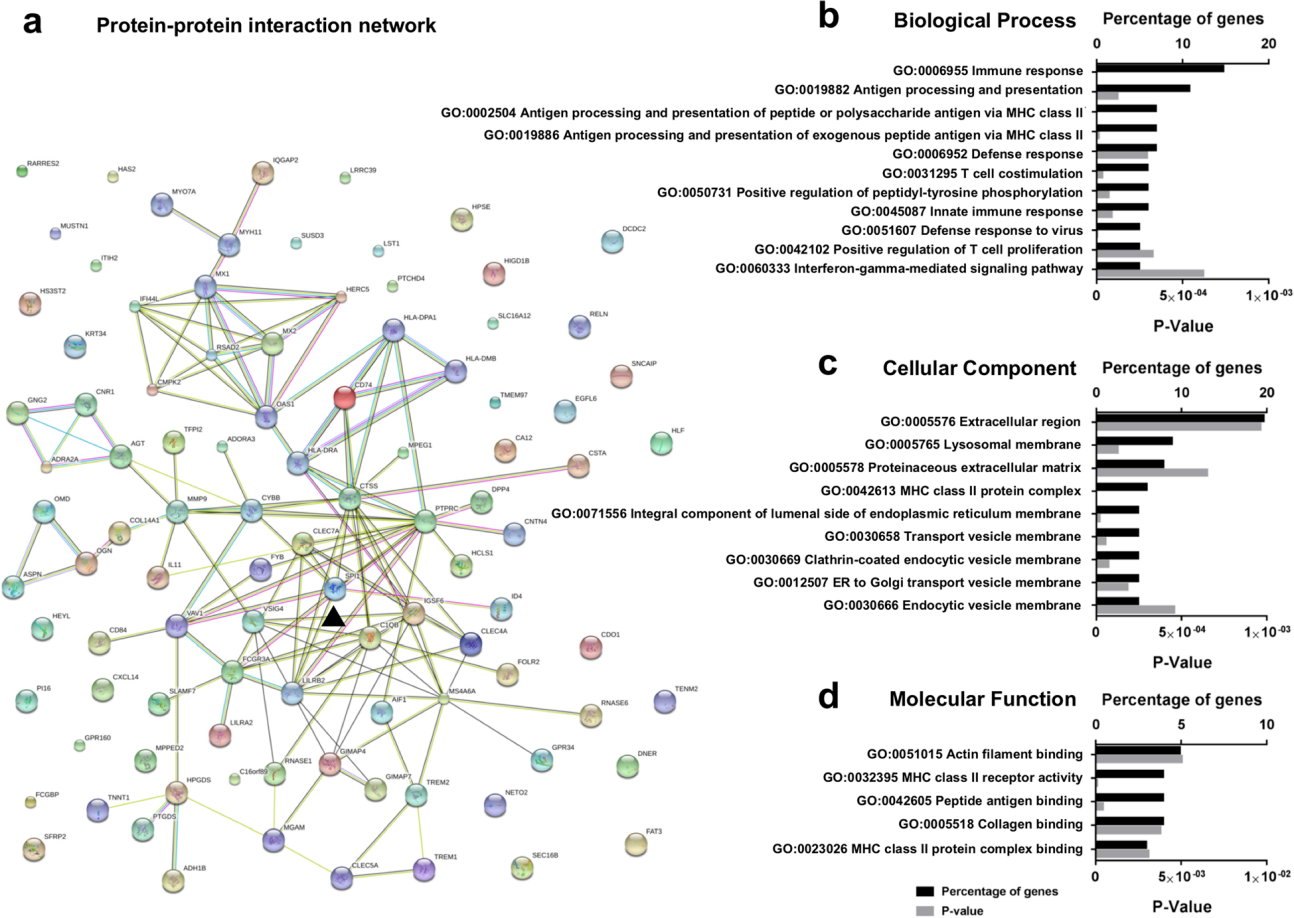
Bioinformatic analysis reveals PU.1 as a highly connected hub protein involved in innate and adaptive immunity. **a** STRING protein-protein interaction network of the top 102 differentially expressed genes specific to mixed glial cultures with PU.1 knockdown implicates PU.1 as a central regulator of altered genes. **b** Gene ontology analysis demonstrating that PU.1-regulated genes are involved in innate and adaptive immune biological processes. **c** The majority of PU.1 regulated genes are localized to the extracellular region, MHCII complexes, or aspects of various endocytic pathways. **d** Molecular functions involved in antigen presentation and actin filament binding were altered by PU.1 silencing

To further examine how PU.1 knockdown modified microglial gene expression in mixed glial cultures several bioinformatic approaches were employed. A STRING protein-protein interaction network of the top 102 differentially expressed genes specific to mixed glial cultures, with inclusion of PU.1 (SPI1), revealed PU.1 as a highly connected hub protein (Fig. 3a). Through gene ontology analysis of differentially regulated genes, PU.1 silencing was found to modify several innate and adaptive biological processes including “immune response”, “antigen processing and presentation”, and “T cell costimulation” (Fig. 3b). Analysis of the cellular components revealed that the majority of modified genes localised to the extracellular region, MHCII protein complexes, and various endocytic vesicle structures (Fig. 3c). Collectively, these revealed changes in molecular functions, including “actin filament binding” and “MHC class II receptor activity” (Fig. 3c) which are implicated in phagocytosis and antigen presentation respectively (Fig. 3d). Lastly, KEGG analysis of differentially expressed genes identified 16 biological pathways, including “phagosome” (Additional file 5: Figure S1) and “antigen processing and presentation” (Additional file 5: Figure S1) containing the largely overlapping genes lists (*CLEC7A*, *FCGR3A*, *CTSS*, *CYBB*, *HLA*-*DMB*, *HLA*-*DPA1*, *HLA*-*DQA1*, *HLA*-*DQB1*, *HLA*-*DR*, and *MRC1*) and (*CD74*, *CTSS*, *HLA*-*DMB*, *HLA-DPA1*, *HLA*-*DQA1*, *HLA*-*DQB1*, and *HLA*-*DRA*) respectively. Additional bioinformatic analysis using GSEA displayed largely similar pathways modified by PU.1 expression (Additional file 6: Table S5).

### Confirmation of microarray analysis in isolated human brain microglia

**Fig. 4:**
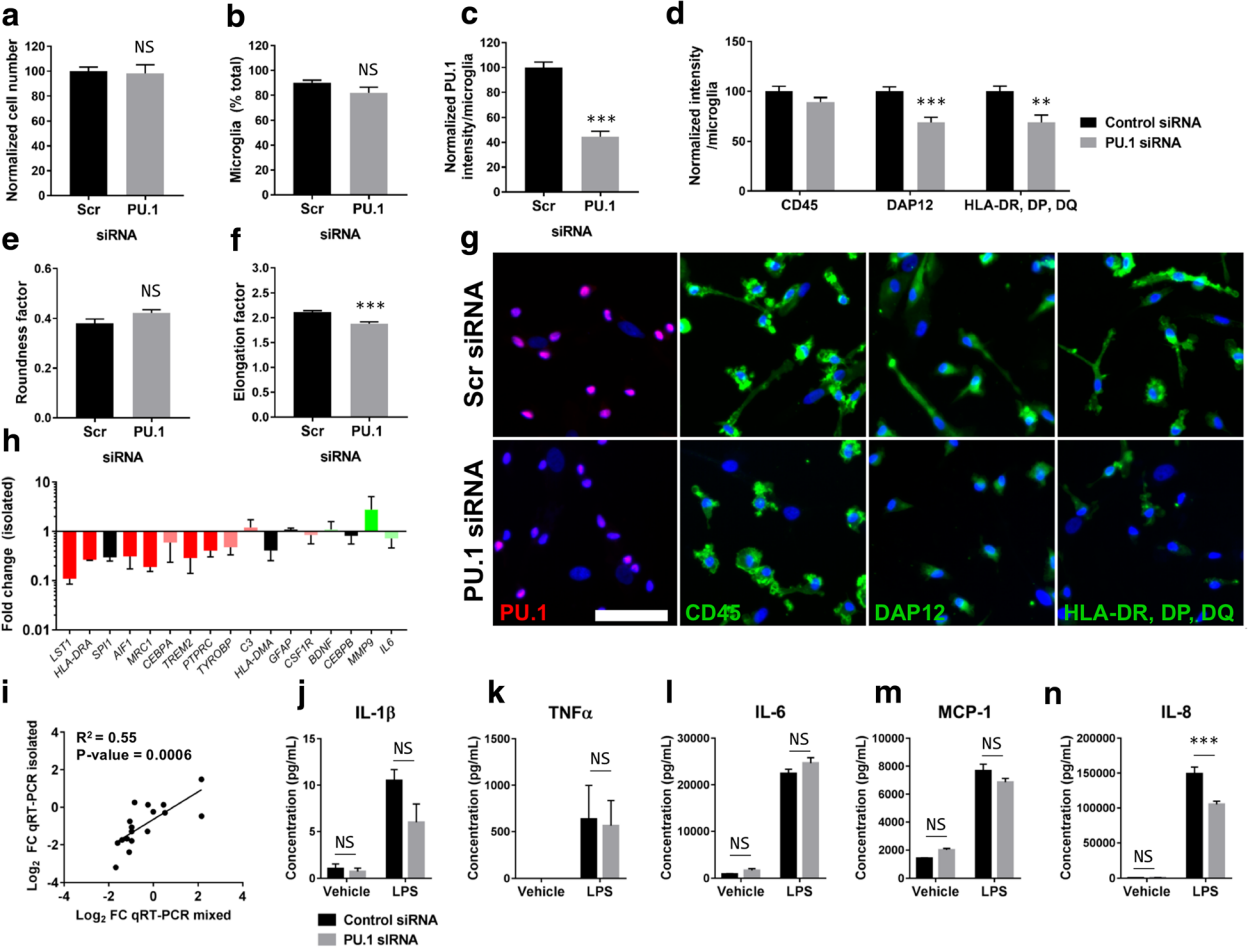
Confirmation of microarray analysis in isolated human brain microglia. **a** Isolated microglia cultures transfected for 7 days with 50 nM PU.1 siRNA show no change in overall cell number (*p* > 0.05), (**b**) no change in the percentage of microglial cells (*p* > 0.05), (**c**) efficient knockdown of PU.1 (*p* < 0.001), (**d**, **g**) reduced expression of DAP12 (*p* < 0.001) and HLA-DR, DP, DQ (*p* < 0.01) but not CD45 (*p* > 0.05), (**e**) non-significant induction in roundness factor (*p* > 0.0.5), and (**f**) reduced elongation factor (*p* < 0.001) compared to a scrambled siRNA control, *n* = 3–5 independent microglial cultures, scale bar = 100 μm. **h** qRT-PCR validation of selected genes in isolated microglia cultures transfected with 50 nM PU.1 siRNA versus control siRNA for 7 days, colour coded by their significance from microarray analysis, (**i**) displayed a significant correlation to changes observed in mixed glial cultures (R^2^ = 0.55), *n* = 3 independent microglial cultures. Data is displayed as fold change of mRNA genes in PU.1 siRNA treated cultures relative to control siRNA samples as determined by the 2^^-ΔΔCt^ method. Cytokine secretions from isolated microglia cultures transfected with 50 nM PU.1 siRNA versus control siRNA for 6 days followed by stimulation with vehicle or 10 ng/mL LPS for a further 24 h demonstrated no effect of PU.1 silencing on LPS-induced (**j**) IL-1β (*p* > 0.05), (**k**) TNFα (*p* > 0.05), (**l**) IL-6 (*p* > 0.05), (**m**) MCP-1 (*p* > 0.05) but a reduction in (**n**) IL-8 (*p* < 0.001). Please note that this is one representative result of three independent experiments. NS = *p* > 0.05, * = *p* < 0.05, ** = *p* < 0.01, *** = *p* < 0.001, Students *t* test

In order to ensure the aforementioned changes in mixed glial cultures were a result of microglial changes with PU.1 silencing and not effects mediated by other cell types, or changes resulting from a gross loss of microglia, further studies were performed using isolated human microglial cultures. Characterisation of isolated microglia with PU.1 knockdown revealed no change in overall cell number (Fig. 4a) or microglial purity (Fig. 4b). Contaminating cells in microglial cultures were identified to be pericytes as described previously [49]. PU.1 siRNA produced a ~ 60% reduction in PU.1 protein expression after 7 days of transfection (Fig. 4c, g). Consistent with gene changes, reductions in DAP12 and HLA-DR, DP, DQ were observed, however there was no change in CD45 (Fig. 4d, g). Analysis of microglial morphology revealed a non-significant increase in rounding (Fig. 4e) and significantly decreased elongation (Fig. 4f). Gene expression, measured by qRT-PCR, reflected changes observed in mixed glial cultures (Fig. 4h, i). Interestingly, overall, inflammatory cytokines and chemokines were largely unaltered in glial cultures with PU.1 knockdown. However, given that gene changes often do not correlate with protein changes, we sought to examine whether PU.1 silencing altered microglial inflammatory cell secretions under both basal and LPS-stimulated conditions. No change was observed in the production of IL-1β (Fig. 4j), TNFα (Fig. 4k), IL-6 (Fig. 4l), or MCP-1 (Fig. 4m), whilst a reduction was observed in IL-8 (Fig. 4n), confirming the lack of effect of PU.1 knockdown on cytokine and chemokine gene expression.

### PU.1-regulated proteins demonstrate microglial expression in the human AD brain

**Fig. 5:**
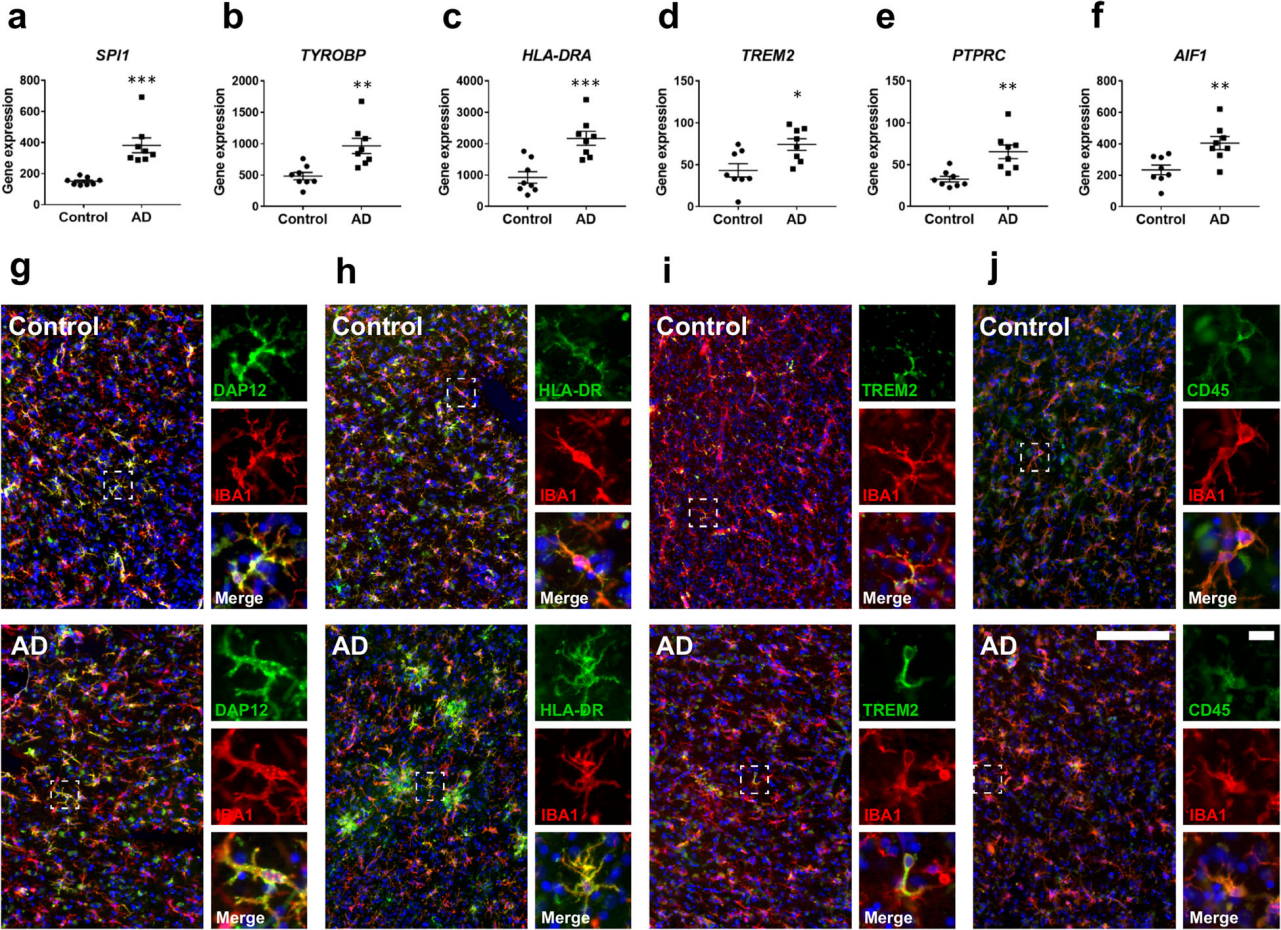
PU.1-regulated proteins demonstrate microglial expression in the human AD brain. NanoString gene expression analysis of selected microglial genes reveals induction of (**a**) *SPI1* (*p* < 0.001), (**b**) *TYROBP* (*p* < 0.001), (**c**) *HLA-DRA* (*p* < 0.001), (**d**) *TREM2* (*p* < 0.05), (**e**) *PTPRC* (*p* < 0.001), and (**f**) *AIF1* (*p* < 0.01) in human brain MFG tissue derived from neurologically normal (*n* = 8) or clinically and pathologically confirmed AD tissue (*n* = 8). Representative images demonstrating microglial localisation of (**g**) DAP12, (**h**) HLA-DR, DP, DQ, (**i**) TREM2, and (**j**) CD45 with IBA1 in the control and AD brain. Scale bar = 200 μm, inset = 20 μm. * = *p* < 0.05, ** = *p* < 0.01, *** = *p* < 0.001, Students *t* test

PU.1 silencing revealed changes in several genes expressed by microglia in vitro. To investigate the relevance of these microglial genes in vivo, RNA was isolated from neurologically normal and pathologically and clinically confirmed AD human brain middle frontal gyrus (MFG) tissue and the expression of several microglial genes was determined by NanoString analysis. An increase in *SPI1* (Fig. 5a), *TYROBP* (Fig. 5b), *HLA-DRA* (Fig. 5c), *TREM2* (Fig. 5d), *PTPRC* (Fig. 5e), and *AIF1* (Fig. 5f) was observed in AD tissue compared to neurologically normal controls, however, this may reflect an alteration in cell populations in the AD brain, including microglial proliferation or neuronal loss, as opposed to changes in individual microglia. Utilising IHC staining, colocalisation of DAP12 (Fig. 5g), HLA-DR, DP, DQ (Fig. 5h), TREM2 (Fig. 5i), and CD45 (Fig. 5j) was observed in IBA1^+^ microglia in both control and AD brains, demonstrating that PU.1-regulated genes are expressed by human microglia in situ.

### High throughput drug screening identified vorinostat as an effective inhibitor of PU.1 that partially mimics the effects of PU.1 silencing

**Fig. 6:**
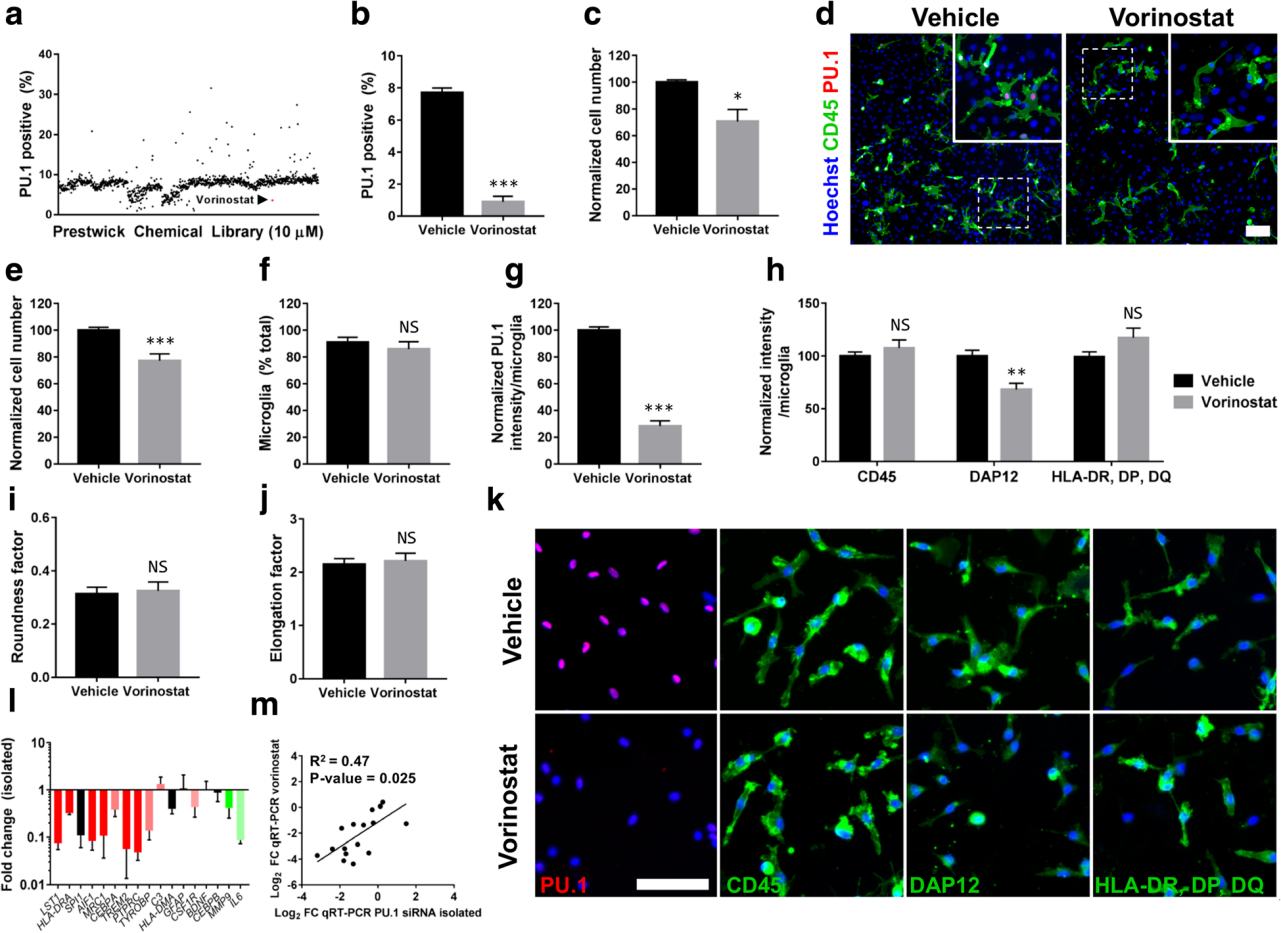
High throughput drug screening identified vorinostat as an effective inhibitor of PU.1 that partially mimics the effects of PU.1 silencing. **a** High throughput drug screening using a drug library containing 1280 FDA-approved compounds at 10 μM for 48 h in mixed glial cultures. ICC and automated image analysis identified the HDAC inhibitor vorinostat as a candidate drug for PU.1 reduction. Please note that this screening experiment was performed using one biological sample. **b** Confirmation of the PU.1 inhibitory effect (*p* < 0.001) of 10 μM vorinostat for 48 h in additional mixed glial cultures analysed by ICC and automated image analysis and (**c**) a mild reduction (*p* < 0.05) in total cell number (**c**), *n* = 2. **d** ICC analysis demonstrating loss of nuclear PU.1 expression with vorinostat treatment in CD45^+^ microglia, scale bar = 100 μm. Isolated microglia cultures treated for 24 h with 10 μM vorinostat show (**e**) a reduction in cell number (*p* < 0.001), (**f**) no change in the percentage of microglial cells (*p* > 0.05), (**g**, **k**) efficient knockdown of PU.1 (*p* < 0.001), (**h**, **k**) reduced expression of DAP12 (*p* < 0.01) but not HLA-DR, DP, DQ (*p* > 0.05) or CD45 (*p* > 0.05), and no change in (**i**) roundness factor or (**j**) elongation factor compared to vehicle treatment, *n* = 3–5, scale bar = 100 μm. (**l**, **m**) Determination of gene changes following a 24 h treatment with 10 μM vorinostat demonstrated a modest correlation (R^2^ = 0.47) with genes altered by PU.1 silencing in isolated microglia cultures, *n* = 3 independent microglial cultures. Data is displayed as fold change of mRNA genes in vorinostat treated cultures relative to vehicle treated samples as determined by the 2^^-ΔΔCt^ method. NS = *p* > 0.05, * = *p* < 0.05, ** = *p* < 0.01, *** = *p* < 0.001, Students *t* test

Having identified that PU.1 silencing modified microglial gene expression involved in antigen presentation/processing and phagocytic functions, we sought to identify potential pharmacological compounds to modify PU.1 expression. Utilising high throughput screening of 1280 compounds (at 10 μM) approved by major regulatory agencies in mixed glial cultures, the HDAC inhibitor vorinostat was found to be highly effective at reducing PU.1 expression (Fig. 6a). Follow up screens in two independent cases confirmed a dramatic reduction in the number of cells displaying PU.1 expression levels compared to vehicle controls (Fig. 6b), whilst a moderate reduction in overall cell number was also observed (Fig. 6c). Further ICC analysis demonstrated that this change was not simply a result of overt microglial loss, as CD45^+^ microglia largely devoid of nuclear PU.1 were observed (Fig. 6d). Further confirmation of this effect was performed in isolated microglia cultures with a 24 h treatment with 10 μM vorinostat which revealed a ~ 20% reduction in total cell number (Fig. 6e) but no alteration in the microglial purity (Fig. 6f). Strong reduction in PU.1 expression by ~ 70% was detected following vorinostat treatment (Fig. 6g, k) and a reduction in DAP12, but not CD45 or HLA-DR, DP, DQ was observed (Fig. 6h, k), partially recapitulating the effects of direct PU.1 silencing with siRNA. No changes in microglial morphology, either through altered elongation (Fig. 6i) or rounding was observed (Fig. 6j). Gene expression analysis by qRT-PCR revealed a moderate correlation with genes altered by siRNA-mediated PU.1 knockdown (Fig. 6l, m).

## Discussion

**Fig. 7:**
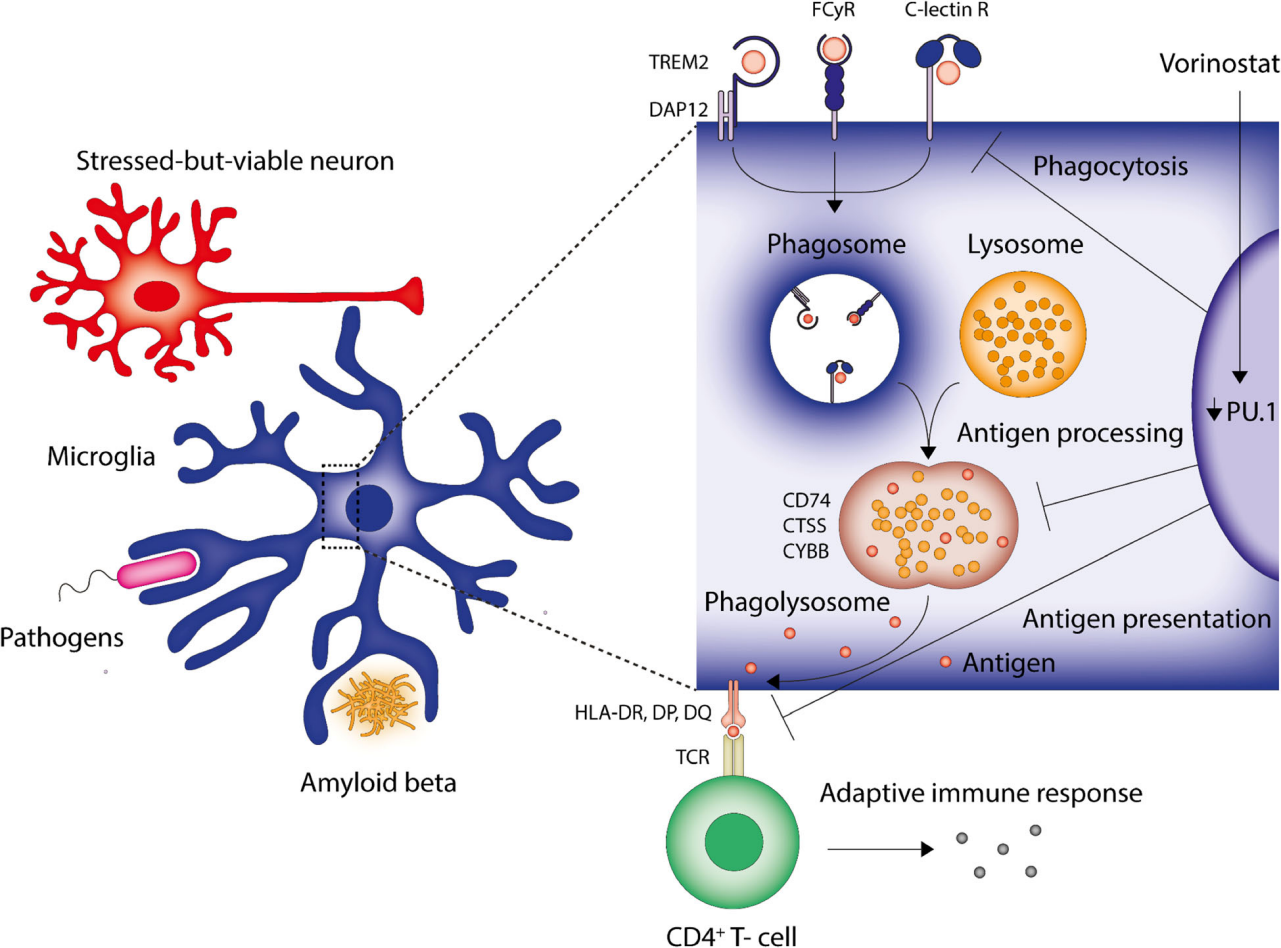
Schematic identifying the contribution of PU.1 to microglial processes. Microglia contain several cell surface receptors implicated in the recognition of various antigenic matter including stressed-but-viable neurons, bacterial and viral pathogens, and misfolded proteins including Aβ_1–42_. PU.1 silencing in microglia attenuated the expression of receptors involved in phagocytic recognition (*TREM2*, *DAP12*, *FCGR3A*, *MRC1*, and *CLEC7A*), antigen processing (CD74, CTSS, and CYBB), and antigen presentation (*HLA*-*DMB*, *HLA-DPA1*, *HLA*-*DQA1*, *HLA*-*DQB1*, and *HLA*-*DRA*) and can be pharmacologically reduced by vorinostat. FCγR = Fc gamma receptor, TCR = T-cell receptor

Microarray and bioinformatic analysis of PU.1 silencing in primary human mixed glial cultures revealed a network of PU.1-regulated genes involved in innate and adaptive immune functions, particularly phagocytic and antigen presentation pathways. These changes were confirmed in isolated cultures of primary human microglia obtained from various neurosurgical samples. Utilising high throughput drug screening of 1280 FDA-approved compounds, the HDAC inhibitor vorinostat was found to attenuate PU.1 expression and mimic several of the changes seen with PU.1 siRNA-mediated silencing. Utilising NanoString analysis of human brain tissue from neurologically normal and pathologically confirmed cases of AD, an induction in *SPI1* and several PU.1-regulated genes, including *TYROBP*, *HLA-DRA*, *TREM2*, *PTPRC*, and *IBA1* was observed. IHC analysis revealed that these markers were all expressed by microglia in neurologically normal and AD brain tissue. Taken together, these data suggest that targeting PU.1 could be beneficial in limiting microglia-mediated immune functions in AD (Fig. 7).

PU.1 is a master regulator of myeloid development and microglial gene expression [33, 34]. Recent evidence suggests that PU.1 also modulates inflammatory responses in rodent BV2 microglia [32] and its attenuation through miRNA124-mediated silencing prevents neuroinflammatory responses in macrophages through reduced MHC-II, TNFα, and iNOS expression [38]. GWAS has identified a common variant in the *CELF1* locus which correlates with reduced PU.1 expression and elevated age of onset for AD, potentially through limiting immune functions [32]. Collectively, these data suggest that mechanisms to reduce PU.1 expression could prove beneficial in limiting microglial-mediated pro-inflammatory contributions in AD. Through ChIP-Seq analysis, several potential target genes for PU.1 have been identified in rodent microglia BV2 cells, including *Csf1r*, *Aif1*, *Trem2,* and *Tyrobp* [52]. Additionally, *Aif1*, *Csf1r*, and *Tyrobp* were found to be attenuated with shRNA-mediated PU.1 knockdown in BV2 cells, implicating these as important genes under PU.1 regulatory control [32]. Importantly, the aforementioned studies were performed in either macrophages or rodent microglia, both of which display unique gene signatures, particularly with respect to several disease-related genes [30, 40, 42]. It is reassuring to confirm several of these changes in human microglia, particularly phagocytosis-related genes including *AIF1*, *TYROBP*, and *TREM2*, in addition to previously unidentified genes. KEGG pathway analysis of differentially regulated genes identified several other genes involved in the phagocytic pathway, including an IgG receptor (*FCGGR3A*), C-lectin receptors (*CLEC7A*, *MRC1*), as well as degradation enzymes of the phagolysosome (*CTSS*) and NADPH oxidase complex (*CYBB*). Altered expression of several genes involved in the antigen processing (*CD74*, *CTSS*) and presentation pathway (*HLA-DMB*, *HLA-DPA1*, *HLA-DQA1*, *HLA-DQB1*, and *HLA-DRA*), a common occurrence following phagocytosis, were also observed. Further, it is critical to ensure that gene changes correspond with altered protein expression. Whilst this was observed with DAP12 and HLA-DR, DP, DQ following PU.1 silencing, CD45 displayed no change, highlighting the importance of confirming these changes when predicting biological functions from microarray or RNA-seq datasets.

Whether a reduction in phagocytosis is likely to be beneficial in AD remains controversial. Removal of parenchymal Aβ plaques has historically been considered to be advantageous through limiting microglial-mediated inflammation, however, whilst recent Aβ immunisation trials were effective in reducing plaque burden, they did not prevent progressive neurodegeneration [53]. Such studies suggest that removal of parenchymal Aβ plaques is not an effective strategy in AD. In contrast, uncontrolled phagocytosis can contribute to inappropriate neuronal removal through phagoptosis, and the TREM2/DAP12 complex has been implicated in this process [54, 55]. Further, inhibition of phagocytosis is sufficient to prevent removal of stressed-but-viable neurons expressing “eat me” signatures, particularly phosphatidylserine, with sub-toxic inflammatory exposures [4, 5, 56–59]. In the AD brain, attenuating phagocytosis/phagoptosis of neuronal cells (or perhaps attenuating synaptic pruning), through PU.1 silencing could prove beneficial in attenuating microglia-mediated neurodegeneration.

Whilst a role for PU.1 in phagocytosis has been previously implicated [33], the contribution of this transcription factor in antigen presentation has been less studied. By virtue of the BBB, the CNS was historically considered an immune privileged site; however, overwhelming evidence now suggests immune surveillance of circumventricular organs, a meningeal lymphatic system, and some degree of parenchymal leucocyte infiltration [60]. As a likely consequence of BBB disruption [61] enhanced extravasation of leucocytes is also observed in the AD brain [62]. In the absence of typical dendritic cells, microglia function as the primary antigen presenting cells (APC) in the brain parenchyma. Whilst the exact extent of their APC capabilities remains somewhat controversial, microglia can be stimulated to express appropriate MHCII (HLA-DR, DP, DQ human equivalent) antigen presentation complexes and contain all required co-stimulatory molecules for appropriate antigen presentation, including CD40, CD80 and CD86, and adhesion molecules including LFA-1 and ICAM-1 [63–65]. Additionally, whilst isolated naïve microglia can activate T-cells, in the brain parenchyma they are likely to be involved in re-stimulation of previously primed T-cells following extravasation, and indeed they are more efficient at this process [65]. Antigen presentation to infiltrated CD4^+^ T-cells resulting in activation or re-priming of CD4^+^ T-cells to a Th1 phenotype could exacerbate inflammatory responses and its attenuation is likely beneficial [62]. Furthermore, it has been suggested that MHCII molecules themselves can function as signal transduction cascades and this MHCII-TCR interaction with T-cells, or indeed other mediators, can promote a pro-inflammatory microglial phenotype [63]. In such instances, preventing the APC properties of microglia may prove advantageous.

We have previously shown that chronic stimulation with the HDAC inhibitor valproic acid (VPA) attenuated human microglial PU.1 expression and AB_1–42_ phagocytosis [66]. Furthermore, its ability to promote neurogenesis and neuroprotection suggested it could prove beneficial in AD patients [67]. However, a randomised control trial utilising VPA was found to accelerate brain atrophy in AD patients with potentially greater cognitive decline [68]. This may be a result of hyper acetylation in the human AD brain [69] which would be further increased by VPA-mediated inhibition of histone deacetylation. Utilising high throughput drug screening of 1280 FDA approved compounds we identified vorinostat, a more potent HDAC inhibitor, to attenuate PU.1 after a single acute exposure at 10 μM. HDAC1 was recently found to activate PU.1 expression by regulating TAF9 deacetylation and IID transcription factor assembly, suggesting that HDAC1 inhibition is an attractive common pathway to modify PU.1 levels [70]. Further, the vorinostat-induced attenuation of DAP12 reflected effects seen in PU.1-knockdown experiments, suggesting that changes may be PU.1-regulated. Whilst vorinostat-altered genes correlated somewhat to PU.1 siRNA in isolated cultures, this was not true for all proteins, including HLA-DR, DP, DQ. Currently, a phase I clinical trial is underway to assess the tolerability of vorinostat in AD patients [71] and whilst modifications in microglial-mediated immune functions are not the primary endpoint of such trials, if found to be tolerable this anti-inflammatory contribution could be beneficial in limiting microglial-mediated neurodegeneration through a PU.1-mediated mechanism. Importantly, vorinostat also displays BBB permeability in both the normal brain and during neurodegenerative disease [72, 73], suggesting appropriate distribution of this drug to the CNS to target microglial-mediated immunity.

Importantly, vorinostat displays broad inhibition of class I and II HDACs enzymes, resulting in widespread gene alterations [74]. As such, vorinostat also displays several PU.1-independent effects, including cell cycle arrest and pro-apoptotic functions [74]. Whilst a reduction in overall cell number was observed, the percentage of microglia remained consistent, suggesting subtle cell type-independent toxicity of 10 μM vorinostat after 24 h. Importantly, the ability to attenuate PU.1 exceeded the toxicity of vorinostat, suggesting that additional titration of vorinostat concentrations could eliminate this toxicity. Further, genetic deletion of PU.1 is embryonically lethal, likely due to aberrant myeloid cell production/differentiation [75], whilst conditional deletion during adulthood [76], as well as significant loss in PU.1 expression in rodents carrying hypomorphic *spi1* alleles [77] precipitated the development of acute myeloid leukemia. As such, any considerations when targeting PU.1 expression should seek simply to attenuate its expression in microglia, as observed with vorinostat treatment, rather than obtain complete removal.

Whilst experiments utilising mixed glial cultures, including microarray analysis, were all performed using cells derived from adult human brain epilepsy tissue, subsequent studies were performed using additional sources of neurosurgical tissue, including various tumour resections and paediatric specimens. This difference, including alterations in age, gender, and disease status could contribute to the partial correlation between isolated and mixed glial cultures gene expression with PU.1 silencing. Such changes could also be explained by alterations in non-microglial cells, including astrocytes and endothelial cells which were present in mixed glial cultures and may respond to changes in microglia functions following PU.1 knockdown. Additionally, microglial gene expression profiles change rapidly after removal from the brain microenvironment and subsequent in vitro culture [30]. Whilst this is an inevitable caveat of in vitro human studies, combining primary human samples and in vivo animal models of PU.1 silencing will be beneficial to fully elucidate the role of PU.1 in microglial-mediated immune functions during neurodegeneration. The finding that attenuating PU.1 expression revealed similar changes independent of the tissue source, and largely the microglial source, is useful in demonstrating the robustness of PU.1 silencing in microglial-modified immune changes.

To investigate whether the aforementioned genes represented valid microglial targets in the human brain, the expression of *SPI1* and several PU.1-regulated genes, *AIF1*, *HLA*-*DRA*, *TREM2*, *TYROBP*, and *PTPRC*, was determined in post mortem neurologically normal and pathologically confirmed AD MFG tissue. All investigated microglial genes demonstrated elevated expression in the AD brain compared to neurologically normal controls, although this could also be a result of altered cell populations in the AD brain. Furthermore, using IHC we show that all tested markers demonstrated exclusively microglial localisation in both the neurologically normal and AD-brain, suggesting that mechanisms to modulate their expression could prove beneficial in limiting microglial phagocytosis and antigen presentation.

Aside from its role in AD risk, PU.1 has been associated with other neurological disorders including Huntington’s disease [35], and hypoxia-ischaemic injury [36]. Microarray analysis in mixed glial cultures revealed that several genes altered by PU.1 silencing were also risk variants [30] for other neurological diseases with a microglial-mediated inflammatory component, including Parkinson’s disease (*HLA*-*DRA*, *HLA*-*DRB5*, *GPNMB*, and *LRRK2*) and multiple sclerosis (*HLA*-*DRB1*, *HLA*-*DRA*, and *CXCR4*) (Additional file 7: Figure S2). Whilst these data should be confirmed by functional protein changes, these findings reinforce the dogma of microglial involvement in numerous neurological disorders and highlight the importance of PU.1 in homeostasis and neurodegeneration.

## Conclusion

Preventing microglial-mediated inflammatory responses is likely to prove favourable in limiting neurodegeneration in a diverse range of neurological disorders, including AD. PU.1 silencing was found to attenuate several genes expressed by microglia in the human brain involved in phagocytic and antigen presentation pathways. High throughput drug screening identified vorinostat as an effective attenuator of microglial PU.1 and this partially recapitulated the effects of siRNA-mediated PU.1 silencing and could prove beneficial in limiting microglial immune contributions to AD pathogenesis.

## Additional files

Additional file 1: **Table S1.** List of cases. List of cases used for all studies. (DOCX 19 kb)

Additional file 2: **Table S2.** List of antibodies and reagents for ICC and IHC (DOCX 17 kb)

Additional file 3: **Table S3.** List of primers used for qRT-PCR (DOCX 18 kb)

Additional file 4: **Table S4.** List of the top 180 differentially expressed genes from microarray analysis (XLSX 27 kb)

Additional file 5: **Figure S1.** KEGG pathway analysis of PU.1 regulated genes. The top 102 uniquely modified genes regulated by PU.1-silencing in mixed glial cultures were subjected to KEGG pathway analysis using DAVID bioinformatics software. Pathways including (a) “Phagosome” and (b) “Antigen Presentation and Processing” were amongst the most changed (c) and reflected the modified genes by Gene Ontology analysis (TIF 32874 kb)

Additional file 6: **Table S5.** GSEA of PU.1 regulated genes. In attempt to identify additional pathways modified by PU.1 silencing in primary human microglia, functionally enriched GO and KEGG gene sets were identified using Gene Set Enrichment Analysis (GSEA) [78, 79] as implemented in WebGestalt [80–82]. The identified pathways (Biological Processes, Molecular Functions, Cellular components, and KEGG analysis) were largely similar to those observed by Fisher’s overrepresentation analysis (Fig. 3b-d), suggesting the significance of PU.1 in the regulation of these functions. (XLSX 28 kb)

Additional file 7: **Figure S2.** PU.1-regulated genes involved in Alzheimer’s disease, Parkinson’s disease and multiple sclerosis risk. A list of risk variants associated with (a) Alzheimer’s disease, (b) Parkinson’s disease, and (c) multiple sclerosis was obtained from [30]. The Log_2_ fold change of these risk variants in PU.1 siRNA versus control siRNA in mixed and pericyte only cultures is displayed. (TIF 32874 kb)

## Abbreviations

AD: Alzheimer’s disease
APC: Antigen presenting cell
Aβ: Amyloid beta
CBA: Cytometric bead array
CNS: Central nervous system
GWAS: Genome wide association studies
HDAC: Histone deacetylase
ICC: Immunocytochemistry
IHC: Immunohistochemistry
MFG: Middle frontal gyrus
MTG: Middle temporal gyrus
qRT-PCR: Quantitative real-time PCR
VPA: Valproic acid
