# Supplementary material for: PU.1 regulates Alzheimer’s disease-associated genes in primary human microglia
Source: Mol Neurodegener. 2018 Aug 20;13:44. doi: 10.1186/s13024-018-0277-1 (PMC6102813; doi:10.1186/s13024-018-0277-1)
Supplement: Supplementary file 1 — Table S1. List of cases. List of cases used for all studies. (DOCX 19 kb) [file 13024_2018_277_MOESM1_ESM.docx]

**Table S1**: List of cases

| **Case** | **Utilisation** | **Experiments** | **Age** | **Sex** | **PMD/PBD** | **COD** | **Pathology** | **Region** |
| --- | --- | --- | --- | --- | --- | --- | --- | --- |
| E179 | Mixed | Microarray, qRT-PCR | 45 | M | 0.5 | NA | Epilepsy | MTG |
| E180 | Mixed | Microarray, qRT-PCR | 53 | M | 0.5 | NA | Epilepsy | MTG |
| E182 | Mixed | Microarray, qRT-PCR | 22 | M | 0.5 | NA | Epilepsy | MTG |
| E202 | Mixed | qRT-PCR | 32 | F | 0.5 | NA | Epilepsy | MTG |
| E205 | Isolated | qRT-pCR | 31 | M | 0.5 | NA | Epilepsy | MTG |
| E208 | Mixed | Drug Screening | 52 | F | 0.5 | NA | Epilepsy | MTG |
| E210 | Mixed | PU.1 siRNA | 30 | M | 0.5 | NA | Epilepsy | MTG |
| E213 | Isolated | Vorinostat | 23 | M | 0.5 | NA | Epilepsy | MTG |
| SS32 | Mixed | Drug Validation | 14 | F | 0.5 | NA | Cortical dysplasia | Unknown |
| SS33 | Mixed | Drug Validation | 7 | M | 0.5 | NA | Cortical tuber | Unknown |
| SS43 | Isolated | PU.1 siRNA,  Vorinostat | 7 | M | 0.5 | NA | Pilocytic astrocytoma | Posterior Fossa |
| SS44 | Isolated | PU.1 siRNA,  Vorinostat | 2 | M | 0.5 | NA | Cortical tuber | MFG |
| SS45 | Isolated | PU.1 siRNA,  Vorinostat | 6 | F | 0.5 | NA | Low Grade Glioma | Temporal lobe |
| T147 | Isolated | PU.1 siRNA,  Vorinostat | 47 | F | 0.5 | NA | Oligodendroglioma | SFG |
| T150 | Isolated | Vorinostat | 44 | F | 0.5 | NA | Glioblastoma multiforme | Unknown |
| H169 | Tissue | IHC | 81 | M | 12 | Asphyxia | No significant histological abnormalities. No Lewy body disease; low Alzheimer’s disease change (A2 B0 C1); cerebral amyloid angiopathy; Tau+ grain-like path in CA1 | MTG |
| H187 | Tissue | IHC | 98 | F | 15 | Caecal carcinoma | Age-related non-specific changes | MTG |
| H196 | Tissue | IHC | 85 | M | 15 | Metastatic Adenocarcinoma | No significant histological abnormalities | MTG |
| H241 | Tissue | IHC | 76 | F | 12 | Metastatic cancer | Alzheimer disease neuropathological change, ABC score: A3 B1 C1; Small vessel hyaline arteriolosclerosis. Normal for age with early Alzheimer’d disease changes. | MTG |
| H243 | Tissue | IHC | 77 | F | 13 | Ischaemic heart disease- coronary atherosclerosis | Normal except infarct occipital blocks | MTG |
| AZ107 | Tissue | IHC | 86 | M | Unknown | Chest infection | Intermediate AD change (A2, B3, C1), Braak VI; Amygdala predominant Lewy body disease; deep small vessel disease; cerebral amyloid angiopathy | MTG |
| AZ108 | Tissue | IHC | 94 | F | 11.5 | Alzheimer’s disease | Consistent with Alzheimer’s disease (NIA-AA score A3 B3 C2, high degree of AD change), LBD, amygdala predominant, cerebral amyloid angiophaty, hyaline arteriolosclerosis | MTG |
| AZ109 | Tissue | IHC | 90 | F | 31 | End stage dementia with no oral intake several days | Consistent with Alzheimer’s disease (NIA-AA score A3 B2 C1, intermediate AD change), cerebral amyloid angiophaty, hyaline arteriolosclerosis | MTG |
| AZ110 | Tissue | IHC | 86 | F | 15 | Severe dementia | Consistent with Alzheimer’s disease (NIA-AA score A3 B3 C2, high AD change); hippocampal sclerosis with associated TDP-43 path; LBD, diffuse; focal cerebral amyloid angiopathy; small vessel disease | MTG |
| H121 | Tissue | NanoString | 64 | F | 6.5 | Pulmonary embolism | No significant histological abnormalities | MFG |
| H122 | Tissue | NanoString | 72 | F | 9 | Emphysema | All unremarkable except hippocampus with agonal haemorrhages; ok as control | MFG |
| H127 | Tissue | NanoString | 59 | F | 21 | Pulmonary embolism | No Alzheimer’s disease change (A0 B0 C0); No transitional or Lewy body disease | MFG |
| H129 | Tissue | NanoString | 48 | M | 12 | Pulmonary embolism | No significant cerebral pathology | MFG |
| H131 | Tissue | NanoString | 73 | F | 13 | Ischaemic heart disease | No significant histological abnormalities | MFG |
| H165 | Tissue | NanoString | 43 | F | 26 | Nitrogen poisoning | No significant histological abnormalities | MFG |
| H170 | Tissue | NanoString | 60 | M | 17 | Ischaemic heart disease | No significant histological abnormalities | MFG |
| H202 | Tissue | NanoString | 83 | M | 14 | Ruptured abdominal aortic aneurysm | No significant changes of degenerative type found. | MFG |
| AZ43 | Tissue | NanoString | 80 | M | 21 | Bronchopneumonia | CERAD: Probable Alzheimer's disease. Atrophy: mild-1, Tangles: mod-2, Plaques: mod-2, ARP: B | MFG |
| AZ45 | Tissue | NanoString | 82 | M | 4.5 | Pneumonia, stroke (6 wks) | CERAD: Probable Alzheimer's disease. Atrophy: mild-1, Tangles: mod-2, Plaques: mod-2, ARP: B | MFG |
| AZ65 | Tissue | NanoString | 77 | F | 16 | Bronchopneumonia | CERAD: Alzheimer's disease (definite). Atrophy: severe, Tangles: mod, Plaques: numerous, ARP: C | MFG |
| AZ71 | Tissue | NanoString | 61 | F | 6 | Severe dementia | CERAD: Definite Alzheimer's disease. Braak: VI; Atrophy: 2/3, Tangles: 3/3, Plaques: 3/3, ARP: C | MFG |
| AZ72 | Tissue | NanoString | 70 | F | 7 | Lung cancer | CERAD: Indicative of Alzheimer's disease. Braak: V; Atrophy: 0/3, Tangles: 1/3, Plaques: 3/3, ARP: C | MFG |
| AZ80 | Tissue | NanoString | 77 | M | 4.5 | Myocardial infarction | CERAD: Definite Alzheimer's disease. Braak: VI; Atrophy: 3/3, Tangles: 3/3, Plaques: 3/3, ARP: C | MFG |
| AZ84 | Tissue | NanoString | 82 | M | 18.5 | Bronchopneumonia | CERAD: probable Alzheimer's disease; Mild Cortical Lewy Body disease. Braak: III, Atrophy: 1/3, Tangles: 1/3, Plaques: 1/3, ARP: A | MFG |
| AZ86 | Tissue | NanoString | 92 | M | 8.5 | Bronchopneumonia; chronic renal failure | CERAD: possible Alzheimer's disease. Braak: III, Atrophy: 0/3, Tangles: 1/3, Plaques: 1/3, ARP: A | MFG |

E = epilepsy, H = neurologically normal, AZ = Alzheimer’s disease, SS = paediatric, T = tumour, PM = post mortem, PB = post biopsy, MTG = middle temporal gyrus, MFG = middle frontal gyrus
