# Supplementary material for: PU.1 regulates Alzheimer’s disease-associated genes in primary human microglia
Source: Mol Neurodegener. 2018 Aug 20;13:44. doi: 10.1186/s13024-018-0277-1 (PMC6102813; doi:10.1186/s13024-018-0277-1)
Supplement: Supplementary file 2 — Table S2. List of antibodies and reagents for ICC and IHC (DOCX 17 kb) [file 13024_2018_277_MOESM2_ESM.docx]

**Table S2**: List of antibodies and reagents for ICC and IHC

| Antibody | Company | Catalogue # | Dilution (ICC) | Dilution (IHC) |
| --- | --- | --- | --- | --- |
| Rabbit anti-PU.1 | Cell Signaling | 2258 | 1:500 | NA |
| Mouse anti-PECAM1 | Dako | MO823 | 1:500 | NA |
| Chicken anti-GFAP | Abcam | Ab4674 | 1:50,000 | NA |
| Rabbit anti-PDGFRβ | Cell Signaling | mAb3169 | 1:500 | NA |
| Mouse anti-CD45 | Abcam | Ab8216 | 1:500 | 1:500 |
| Rabbit anit-DAP12 | Santa Cruz | SC-20783 | 1:500 | 1:100 |
| Mouse anti-HLA-DR, DP, DQ | DAKO | MO775 | 1:500 | 1:500 |
| Goat anti-TREM2 | R&D Systems | AF1828 | NA | 1:500 |
| Goat anti-IBA1 | Abcam | Ab5076 | NA | 1:1,000 |
| Rabbit anti-IBA1 | Abcam | Ab178846 | NA | 1:1,000 |
| Alexa Fluor® 488 Goat anti-Mouse | Invitrogen | A11001 | 1:500 | NA |
| Alexa Fluor® 594 Goat anti-Rabbit | Invitrogen | A11012 | 1:500 | NA |
| Alexa Fluor® 647 Donkey anti-Chicken | Invitrogen | A21449 | 1:500 | NA |
| Alexa Fluor® 488 Donkey anti-Goat | Invitrogen | R37114 | NA | 1:250 |
| Alexa Fluor®594 Donkey anti-Mouse | Invitrogen | R37115 | NA | 1:250 |
| Biotinylated Donkey anti-Rabbit | Jackson Labs | 711-065-112 | NA | 1:250 |
| Alexa Fluor® 594 Donkey anti-Rabbit | Invitrogen | R37119 | NA | 1:250 |
| Alexa Fluor® 647 Donkey anti-Rabbit | Invitrogen | A31573 | NA | 1:250 |
| Biotinylated Donkey anti-Goat | Jackson Labs | 705-065-003 | NA | 1:250 |
| Alexa Fluor® 594 Donkey anti-Goat | Invitrogen | A11058 | NA | 1:250 |
| Alexa Fluor® 647 Donkey anti-Goat | Invitrogen | A21447 | NA | 1:250 |
| Extravidin peroxidase | Sigma Aldrich | E2886 | NA | 1:500 |
|  |  |  |  |  |
