# Supplementary material for: PU.1 regulates Alzheimer’s disease-associated genes in primary human microglia
Source: Mol Neurodegener. 2018 Aug 20;13:44. doi: 10.1186/s13024-018-0277-1 (PMC6102813; doi:10.1186/s13024-018-0277-1)
Supplement: Supplementary file 3 — Table S3. List of primers used for qRT-PCR (DOCX 18 kb) [file 13024_2018_277_MOESM3_ESM.docx]

**Table S3**: List of primers used for qRT-PCR

| Gene | Protein |  | Sequence | Amplicon Length |
| --- | --- | --- | --- | --- |
| *CEBPA* | CEBPα | FW | GCCGGGAGAACTCTAACTCC | 82 |
|  |  | RV | TGCAGGTGGCTGCTCAT |  |
| *CEBPB* | CEBPβ | FW | AGAGCAAGGCCAAGAAGAC | 77 |
|  |  | RV | CACGGCGATGTTGTTGC |  |
| *C3* | C3 | FW | GAGGACGAATGCCAAGACGA | 72 |
|  |  | RV | AACCATGCTCTCGGTGAAGG |  |
| *SPI1* | PU.1 | FW | CACGGATCTATACCAACGCCA | 82 |
|  |  | RV | CCAGTAATGGTCGCTATGGC |  |
| *TREM2* | TREM2 | FW | GGGAGTCTGAGAGCTTCGAG | 84 |
|  |  | RV | TGGGTGGGAAGGGGATTTCT |  |
| *IL6* | IL-6 | FW | TTCGGTCCAGTTGCCTTCTC | 80 |
|  |  | RV | TCTTCTCCTGGGGGTACTGG |  |
| *HLA-DMA* | HLA-DMA | FW | ACGACGAGGACCAGCTTTTC | 81 |
|  |  | RV | GAGCCCAGTCAGCAAATTCG |  |
| *GFAP* | GFAP | FW | TGACCGCTTTGCCAGCTACATCG | 69 |
|  |  | RV | TCAGCAGCCAGCGCCTTGTTT |  |
| *AIF1* | IBA1 | FW | TGTCTCCCCACCTCTACCAG | 85 |
|  |  | RV | CCTTCAGCAGTCCGAAAGCT |  |
| *BACTIN* | Beta actin | FW | TGGTGGGCATGGGTCAGAAGGA | 94 |
|  |  | RV | ATGCCGTGCTCGATGGGGTACT |  |
| *PTPRC* | CD45 | FW | TGTGGCTTAAACTCTTGGCAT | 77 |
|  |  | RV | AGGTGTTGGGCTTTGCC |  |
| *CSF1R* | M-CSFR | FW | CCAGGCTAAAAGGGGAAGAAG | 85 |
|  |  | RV | CGTTCCTCTCCTCTGCACT |  |
| *TYROBP* | DAP12 | FW | ACCCGGAAACAGCGTATCAC | 70 |
|  |  | RV | AGACATCCGACCTCTGACCC |  |
| *HLA-DRA* | HLA-DRA | FW | AGCCCTGTGGAACTGAGAGA | 84 |
|  |  | RV | AAGCCACGTGACATTGACCA |  |
| *LST1* | LST1 | FW | GGACTAGAGTTCCTGACCTCC | 85 |
|  |  | RV | GCTCCTCTCCAGCCTCTTTAC |  |
| *MMP9* | MMP9 | FW | ATGTACCGCTTCACTGAGGG | 81 |
|  |  | RV | TTCAGGGCGAGGACCATAGA |  |
| *MRC1* | MRC1 | FW | TGGATGGATGATACCTGCGAC | 70 |
|  |  | RV | TAGTCAAGGAAGGGTCGGATC |  |
| *GAPDH* | GAPDH | FW | CATGAGAAGTATGACAACAGCCT | 113 |
|  |  | RV | AGTCCTTCCACGATACCAAAGT |  |
| *BDNF* | BDNF | FW | TGGATGAGGACCAGAAAGTTCGGC | 80 |
|  |  | RV | ACTGAGCATCACCCTGGACGTGT |  |
