# Supplementary figures and images for: PU.1 regulates Alzheimer’s disease-associated genes in primary human microglia
Source: Mol Neurodegener. 2018 Aug 20;13:44. doi: 10.1186/s13024-018-0277-1 (PMC6102813; doi:10.1186/s13024-018-0277-1)

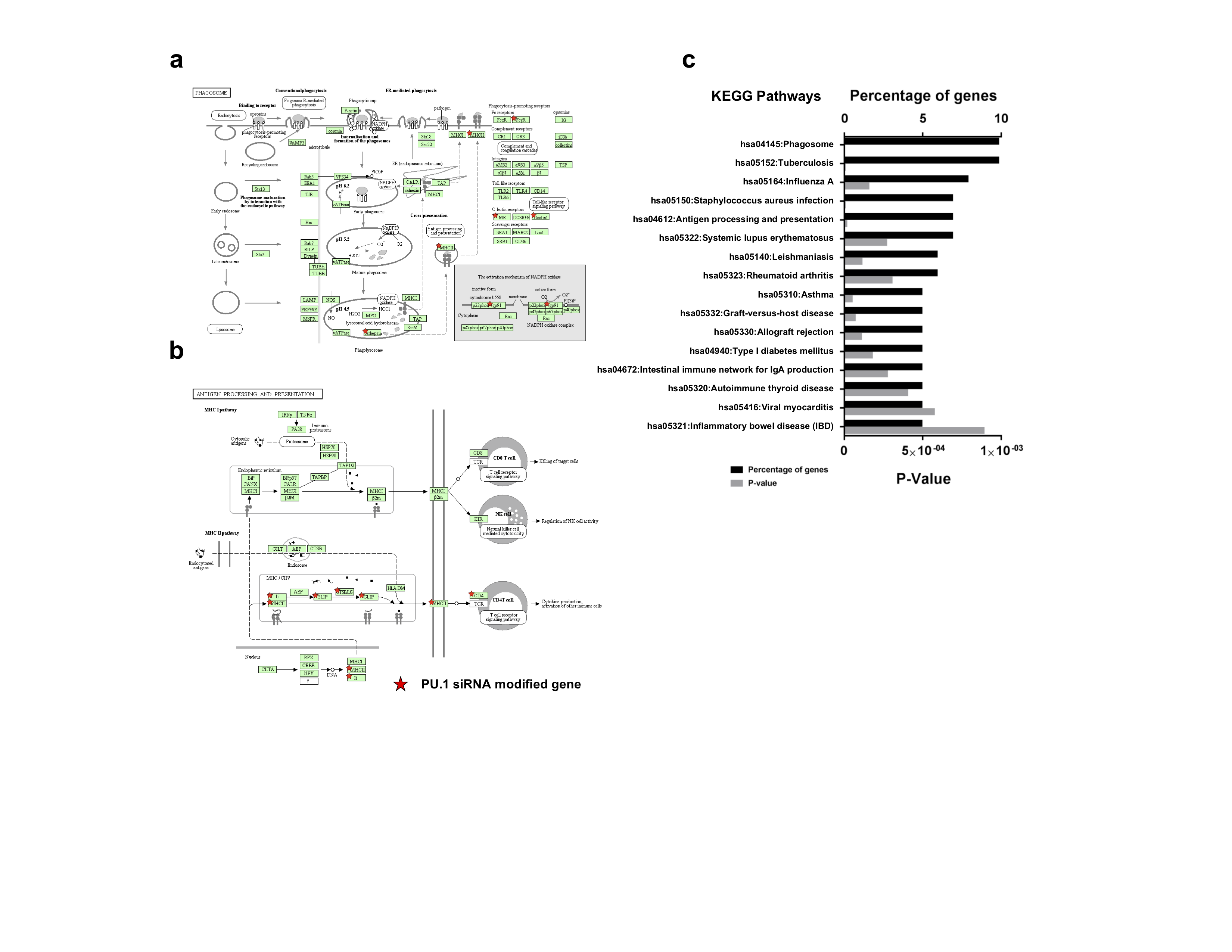

Supplement: Supplementary file 5 — Figure S1. KEGG pathway analysis of PU.1 regulated genes. The top 102 uniquely modified genes regulated by PU.1-silencing in mixed glial cultures were subjected to KEGG pathway analysis using DAVID bioinformatics software. Pathways including (a) “Phagosome” and (b) “Antigen Presentation and Processing” were amongst the most changed (c) and reflected the modified genes by Gene Ontology analysis (TIF 32874 kb) [file 13024_2018_277_MOESM5_ESM.tif]

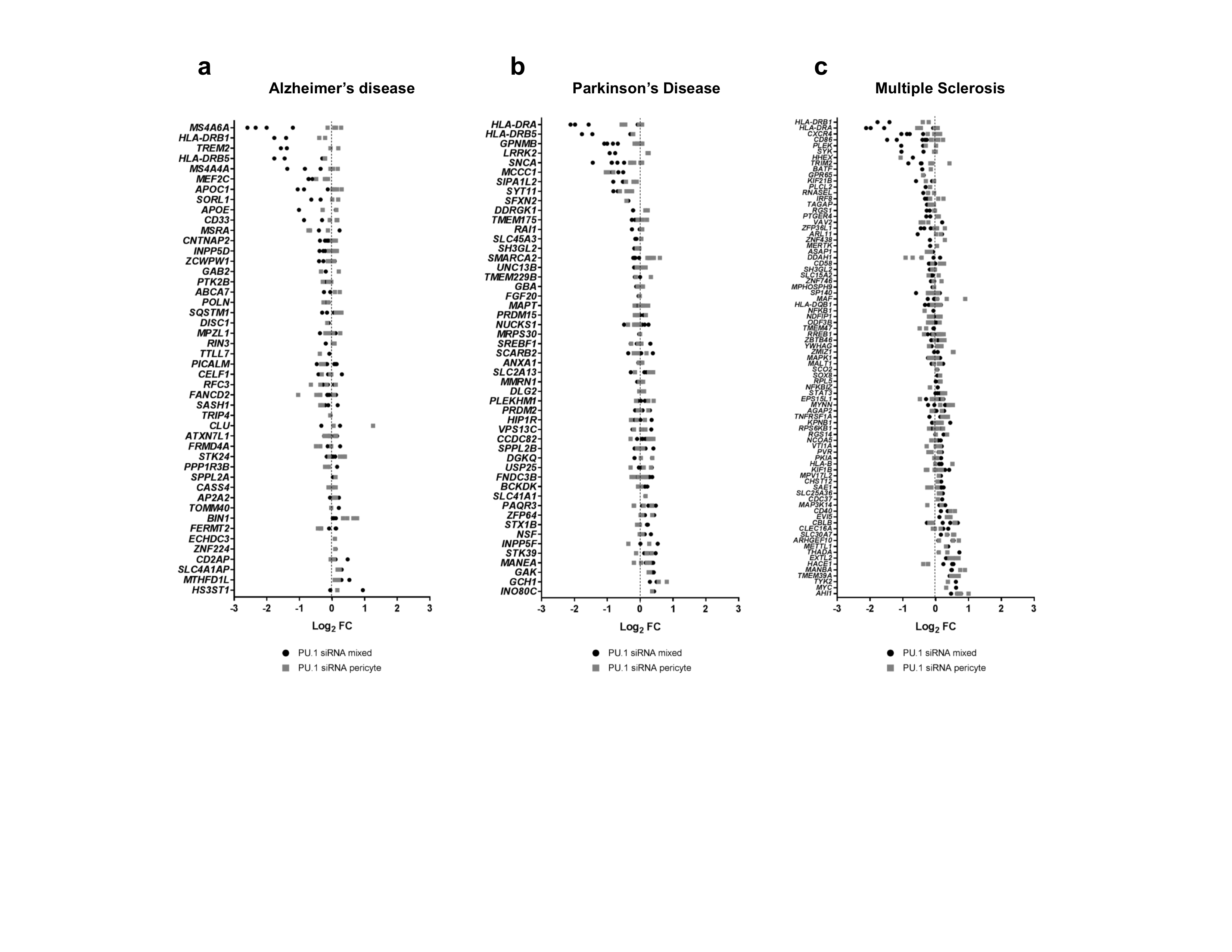

Supplement: Supplementary file 7 — Figure S2. PU.1-regulated genes involved in Alzheimer’s disease, Parkinson’s disease and multiple sclerosis risk. A list of risk variants associated with (a) Alzheimer’s disease, (b) Parkinson’s disease, and (c) multiple sclerosis was obtained from [30]. The Log2 fold change of these risk variants in PU.1 siRNA versus control siRNA in mixed and pericyte only cultures is displayed. (TIF 32874 kb) [file 13024_2018_277_MOESM7_ESM.tif]
